# Hyperammonemia syndrome due to *Pluralibacter gergoviae* bacteremia in an immunocompromised patient: case report

**DOI:** 10.1128/asmcr.00215-25

**Published:** 2026-01-08

**Authors:** Rami Waked, Jonathan Huang, Lucy S. Witt, Eileen M. Burd

**Affiliations:** 1 Department of Pathology and Laboratory Medicine, Emory University School of Medicine 12239, Atlanta, Georgia, USA; 2 Department of Medicine, Division of Infectious Diseases, Emory University School of Medicine 12239, Atlanta, Georgia, USA; Rush University Medical Center, Chicago, Illinois, USA

**Keywords:** *Pluralibacter gergoviae*, hyperammonemia, immunocompromised host, transplant, bacteremia

## Abstract

**Background:**

*Pluralibacter gergoviae* is a rare, opportunistic, urease-producing gram-negative organism infrequently implicated in invasive infections. Reports in organ transplant or extracorporeal membrane oxygenation (ECMO) settings are exceedingly uncommon. This case is distinctive for describing *P. gergoviae* bacteremia potentially associated with severe hyperammonemia and encephalopathy in a lung transplant recipient on prolonged ECMO support, expanding the clinical spectrum of infection-related hyperammonemia beyond the traditionally recognized *Ureaplasma* and *Mycoplasma* species.

**Case Summary:**

A 32-year-old woman with cystic fibrosis underwent bilateral lung transplantation while on ECMO for refractory hypoxemic respiratory failure. Her post-operative course was complicated by multiorgan failure, *Candida parapsilosis* fungemia, and later, surgical site infection. During the fourth month of hospitalization, *P. gergoviae* was isolated from surgical wound and blood cultures. Around the time of bacteremia, she developed acute encephalopathy with markedly elevated plasma ammonia (262 µmol/L) in the absence of hepatic dysfunction. The *P. gergoviae* isolate was confirmed to be urease positive. Management included targeted antimicrobial therapy with ceftriaxone and meropenem, along with lactulose, rifaximin, and zinc for hyperammonemia. Ammonia levels and mental status gradually normalized following treatment, and blood cultures cleared.

**Conclusion:**

This case illustrates *P. gergoviae* as an emerging opportunistic pathogen capable of causing invasive infection and possibly metabolic complications in profoundly immunocompromised hosts.

## INTRODUCTION

*Pluralibacter gergoviae* is a gram-negative, oxidase-negative, nitrate-positive, urease-positive, fermentative, rod-shaped organism belonging to the family Enterobacteriaceae (1, 2). Formerly classified as *Enterobacter gergoviae* due to its biochemical similarity to *Enterobacter* species, it has been reclassified into the genus *Pluralibacter* and is now designated as *P. gergoviae* (1). This organism has been identified as a contaminant in cosmetic products, as a colonizer of the human oral cavity, and as a cause of nosocomial outbreaks and bloodstream infections (3–8).

Hyperammonemia syndrome is a life-threatening complication in lung transplant recipients characterized by elevated serum ammonia and altered mentation. *Mycoplasma* and *Ureaplasma* infections are well-established contributors through ammonia overproduction (9–11). While not as common, the association of hyperammonemia syndrome with urease-producing Enterobacterales has been increasingly recognized (9, 12–16).

## CASE PRESENTATION

A woman in her 30s with a history of cystic fibrosis complicated by chronic hypoxemic respiratory failure requiring home supplemental oxygen and pancreatic insufficiency was admitted to the hospital with acute hypoxemic respiratory failure necessitating endotracheal intubation upon arrival to the emergency department. Her medical history was notable for recurrent pulmonary infections with *Pseudomonas aeruginosa* and methicillin-resistant *Staphylococcus aureus* and chronic malnutrition requiring percutaneous gastrostomy tube placement.

### Initial hospital course

A respiratory pathogen PCR panel (BIOFIRE Respiratory 2.1 Panel; bioMérieux, Durham, NC) was positive for respiratory syncytial virus and rhinovirus. Tracheal aspirate culture yielded methicillin-susceptible *S. aureus*, prompting the use of intravenous cefazolin 2 g every 8 hours, and inhaled tobramycin was continued for prior *P. aeruginosa*.

Despite targeted therapy, the patient remained profoundly hypoxemic and mechanical ventilator dependent. She was transitioned to veno-venous extracorporeal membrane oxygenation (ECMO) and eventually underwent an urgent off-pump bilateral orthotopic lung transplant around 6 weeks after admission. The patient was cytomegalovirus and Epstein–Barr virus seropositive. She was started on an immunosuppressive regimen with methylprednisolone, tacrolimus, and basiliximab. She also received atovaquone 1,500 mg once daily, inhaled amphotericin B, ganciclovir 5 mg/kg every 24 hours, and posaconazole 300 mg daily prophylaxis.

### Post-operative course

The post-operative period was marked by significant complications. The patient developed low ECMO flow state necessitating conversion to central veno-arterial ECMO cannulation. Subsequently, she experienced intra-abdominal compartment syndrome with bowel ischemia, multiorgan failure, and an expanding retroperitoneal hematoma, which required lumbar artery embolization.

During this time, imaging demonstrated an evolving right lower lobe cavitary consolidation consistent with lung abscess, as well as a persistent right pleural air leak. Chest computed tomography revealed findings concerning for a bronchopleural fistula, for which the patient underwent operative intervention. Her course was further complicated by severe acute kidney injury requiring initiation of renal replacement therapy.

### Late hospital course

**Fig 1:**
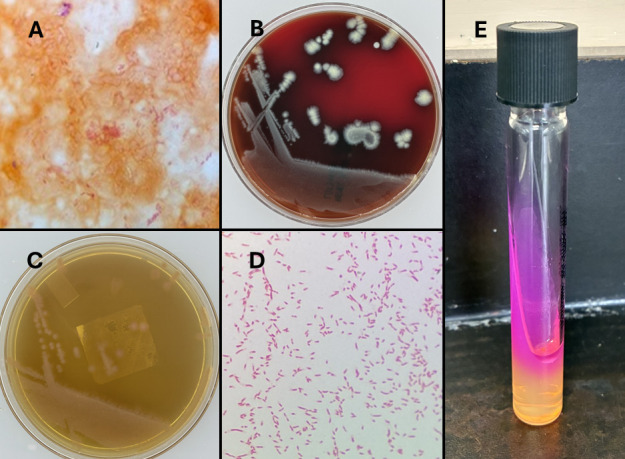
(**A**) Gram stain of positive blood culture showing gram-negative bacilli. (**B and C**) *Pluralibacter gergoviae* growth on blood agar and MacConkey agar, respectively, at 24 hours of incubation. (**D**) Gram stain from colonies on culture media showing gram-negative bacilli and some coccobacilli. (**E**) Positive urease test showing pink-red coloration change, indicating an alkaline change.

At the fourth to fifth month of hospitalization, the patient remained on ECMO support. Her initial surgical wound became infected with cultures growing *P. gergoviae*, *Candida parapsilosis*, and vancomycin-resistant *Enterococcus*. Blood cultures obtained later grew *P. gergoviae* in one of two bottles after approximately 46 hours of incubation (Fig. 1A through D). Rapid identification using the BIOFIRE BCID2 PCR panel (bioMérieux) detected Enterobacterales, and species-level identification of *P. gergoviae* was achieved by matrix-assisted laser desorption/ionization time-of-flight (MALDI-TOF) mass spectrometry (confidence level 99.9%, bioMérieux). Antimicrobial susceptibility testing on the VITEK 2 system (bioMérieux) demonstrated resistance to cefpodoxime and cefazolin and dose-dependent susceptibility to piperacillin-tazobactam (see Table 1 for complete susceptibility results). This isolate was confirmed to be urease positive (Fig. 1E).

**TABLE 1:** *Pluralibacter gergoviae* susceptibility testing result

Antibiotic	MIC (μg/mL)	Interpretation
Cefazolin	≥32.0	Resistant
Cefepime	2.0	Susceptible
Cefpodoxime	≥8.0	Resistant
Ceftazidime	4.0	Susceptible
Ceftazidime + avibactam	2.0	Susceptible
Ceftriaxone	0.5	Susceptible
Ciprofloxacin	≤0.06	Susceptible
Ertapenem	≤0.12	Susceptible
Levofloxacin	≤0.12	Susceptible
Meropenem	≤0.25	Susceptible
Piperacillin + tazobactam	16.0	Susceptible-dose dependent
Tobramycin	≤1.0	Susceptible
Trimethoprim + sulfamethoxazole	≤20.0	Susceptible

### Hyperammonemia episode

Around the time of the *P. gergoviae* bacteremia, the patient developed acute encephalopathy. Laboratory evaluation revealed a markedly elevated plasma ammonia level of 262 µmol/L (reference range ≤53 µmol/L), consistent with hyperammonemia syndrome. At the time, she was receiving intravenous eravacycline 1 mg/kg every 12 hours, isavuconazole 372 mg daily, and meropenem 1g every 8 hours for her infections. There was no radiologic or clinical evidence of hepatic cirrhosis. Thyroid function tests demonstrated subclinical hypothyroidism. The neurocritical care service was consulted, and non-hepatic causes of hyperammonemia were considered, including infection-related hyperammonemia secondary to *P. gergoviae* bacteremia, medication-induced effects (tacrolimus), and nutritional factors. Molecular testing for Mollicutes (*Mycoplasma* and *Ureaplasma*) was performed using multiple modalities. *Mycoplasma pneumoniae* was assessed on the bronchoalveolar lavage (BAL) specimen using the in-house BIOFIRE FILMARRAY Pneumonia Panel (bioMérieux), which was negative. In addition, a *Ureaplasma*/*Mycoplasma* PCR was performed on the BAL at ARUP Laboratories (Salt Lake City, UT), and this also yielded negative results.

Therapeutic management included oral rifaximin 400 mg three times daily, lactulose two to three times daily, acetohydroxamic acid, and zinc supplementation, in addition to antibiotics. Serial monitoring showed gradual normalization of ammonia levels, accompanied by improvement in mental status and resolution of encephalopathy within 2 weeks from initiation of targeted antibiotic therapy. Repeat blood cultures in the subsequent days did not show any bacterial growth. Two weeks of antibiotic therapy were completed in total. Based on susceptibility results, meropenem was discontinued, and ceftriaxone 2g IV daily was initiated; however, due to subsequent hemodynamic instability, renally dosed meropenem was reintroduced. These adjustments reflect how antimicrobial agents (including dosage and selection) were modified in direct response to the *P. gergoviae* culture result and evolving clinical status.

## DISCUSSION

*P. gergoviae* has been implicated as a causative pathogen in multiple clinical syndromes, including bacteremia (1–3), pulmonary infection (4, 5), urinary tract infection (4, 6), and endophthalmitis (7). In the present case, *P. gergoviae* was isolated from both surgical wound and bloodstream cultures in a lung transplant recipient. Distinguishing between colonization and true infection with this organism can be challenging, particularly in critically ill or immunocompromised hosts; however, the temporal association of positive cultures with clinical deterioration and compatible infectious findings in this patient supported its pathogenic role rather than colonization. The use of modern molecular diagnostic tools, including MALDI-TOF mass spectrometry and multiplex blood culture identification panel, was essential for the rapid and accurate identification of this uncommon Enterobacterales species. Infections caused by this organism have been most frequently described in transplant recipients (4, 6), patients with hematologic malignancies (3), and in neonatal outbreak settings (1, 2).

Hyperammonemia syndrome has been reported as a post-lung transplant complication, most often attributed to *Ureaplasma* or *Mycoplasma* species infections of the allograft (10). In a single-center study, the prevalence of hyperammonemia syndrome was 3.9% in lung transplant patients compared to 0.1% in other solid organ recipients (9). While this complication occurs with a mean onset of 11 days post-transplantation (11), hyperammonemia developed approximately 4 months after transplant in this recipient. Recognizing infection-associated hyperammonemia is crucial in critically ill and transplant patients, as early treatment of the infectious source and prompt metabolic management are key to preventing neurologic complications and improving outcomes.

In this case, *P. gergoviae* bacteremia was temporally associated with the onset of marked hyperammonemia and encephalopathy in the absence of hepatic dysfunction or mollicute detection, suggesting a possible contribution to the metabolic disturbance. However, molecular assays have imperfect sensitivity and do not uniformly detect all *Ureaplasma* and *Mycoplasma* species. Notably, the patient was receiving eravacycline at the time of testing, which has excellent activity against Mollicutes, further reducing the likelihood of an undetected mollicute infection. *P. gergoviae* is a urease-producing organism, confirmed by a positive urease test in this case (Fig. 1E) (8). The enzymatic breakdown of urea by urease generates ammonia and carbon dioxide as byproducts, leading to increased systemic ammonia levels. Additional contributing factors may include the use of immunosuppressive agents such as tacrolimus and cyclosporine, which have been shown to alter the expression of genes responsible for urea clearance (16, 17).

*P. gergoviae* demonstrates variable susceptibility to β-lactams, fluoroquinolones, aminoglycosides, and tetracycline derivatives (18). Reported resistance mechanisms include extended-spectrum β-lactamases and carbapenemases such as NDM, IMP, and KPC, underscoring the importance of antimicrobial susceptibility testing for definitive therapy (1, 3, 4, 6, 19). In this case, the isolate was susceptible (except for resistance to cefpodoxime and cefazolin), which supported de-escalation from empiric broad-spectrum coverage (meropenem) to a targeted regimen (ceftriaxone) guided by *in vitro* data.

Although reports of *P. gergoviae* infection in transplant or ECMO patients are rare, a few prior cases offer useful context. One similar case described colonization with *P. gergoviae* in a recent lung transplant recipient (4). In another case series, *P. gergoviae* outbreak was described among kidney transplant recipients (6). Separately, a case report of septic shock caused by KPC-producing *E. gergoviae* (i.e., prior nomenclature) in a neutropenic patient with leukemia illustrates the ability of the organism to cause fulminant invasive disease in immunocompromised hosts (3). Compared to these prior reports, our case is distinctive in several ways: it involves a lung transplant recipient on prolonged ECMO support, and the isolate was pan susceptible rather than carbapenemase harboring.

This case highlights *P. gergoviae* as an emerging opportunistic pathogen capable of causing invasive infection and possibly metabolic complications in profoundly immunocompromised hosts, including lung transplant recipients supported with ECMO. The temporal association between *P. gergoviae* bacteremia and marked hyperammonemia in the absence of hepatic dysfunction underscores the importance of recognizing infection-associated hyperammonemia, particularly with urease-producing organisms.
